# Methanol loading dependent methoxylation in zeolite H-ZSM-5†
†Electronic supplementary information (ESI) available. See DOI: 10.1039/d0sc01924k


**DOI:** 10.1039/d0sc01924k

**Published:** 2020-06-17

**Authors:** Santhosh K. Matam, Stefan A. F. Nastase, Andrew J. Logsdail, C. Richard. A Catlow

**Affiliations:** a UK Catalysis Hub, Research Complex at Harwell , Science and Technology Facilities Council , Rutherford Appleton Laboratory , Oxford , OX11 0FA , UK . Email: santhosh.matam@rc-harwell.ac.uk ; [http://www.ukcatalysishub.co.uk/]; b Cardiff Catalysis Institute , School of Chemistry , Cardiff University , Cardiff , CF10 3AT UK; c Department of Chemistry , University College London , 20 Gordon St. , London WC1E 6BT , UK

## Abstract

We evaluate the effect of the number of methanol molecules per acidic site of H-ZSM-5 on the methoxylation reaction at room temperature by applying *operando* diffuse reflectance infrared Fourier transformed spectroscopy (DRIFTS) and mass spectrometry (MS), which capture the methoxylation reaction by simultaneously probing surface adsorbed species and reaction products, respectively. To this end, the methanol loading in H-ZSM-5 (Si/Al ≈ 25) pores is systematically varied between 32, 16, 8 and 4 molecules per unit cell, which corresponds to 8, 4, 2 and 1 molecules per Brønsted acidic site, respectively. The *operando* DRIFTS/MS data show that the room temperature methoxylation depends on the methanol loading: the higher the methanol loading, the faster the methoxylation. Accordingly, the reaction is more than an order of magnitude faster with 8 methanol molecules per Brønsted acidic site than that with 2 molecules, as evident from the evolution of the methyl rock band of the methoxy species and of water as a function of time. Significantly, no methoxylation is observed with ≤1 molecule per Brønsted acidic site. However, hydrogen bonded methanol occurs across all loadings studied, but the structure of hydrogen bonded methanol also depends on the loading. Methanol loading of ≤1 molecule per acidic site leads to the formation of hydrogen bonded methanol with no proton transfer (*i.e.* neutral geometry), while loading ≥2 molecules per acidic site results in a hydrogen bonded methanol with a net positive charge on the adduct (protonated geometry). The infrared vibrational frequencies of methoxy and hydrogen bonded methanol are corroborated by Density Functional Theory (DFT) calculations. Both the experiments and calculations reflect the methoxy bands at around 940, 1180, 2868–2876 and 2980–2973 cm^–1^ which correspond to *ν*(C–O), *ρ*(CH_3_), *ν*_s_(C–H) and *ν*_as_(C–H), respectively.

## Abstract 2

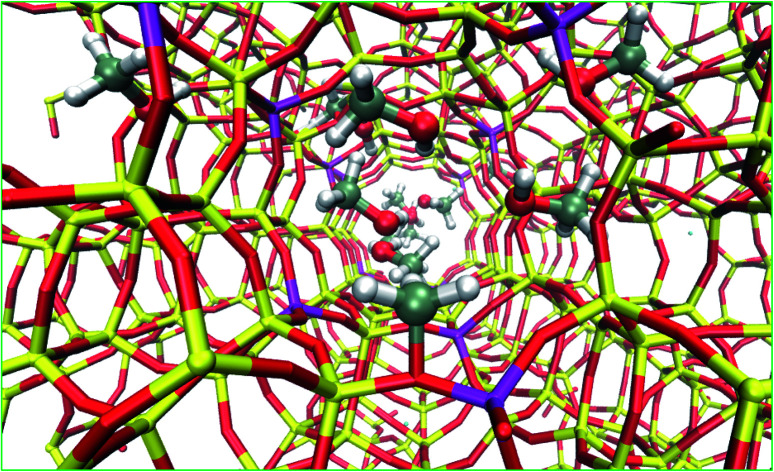
Room temperature methoxylation is methanol loading dependent: the higher the methanol loading, the faster the methoxylation. Methanol load of ≥2 leads to methoxylation while no methoxylation is observed with ≤1 molecule per Brønsted acidic site.

## Introduction

A myriad of industrial catalytic processes, ranging from petro- and fine-chemical to environmental processes, depend on crystalline zeolites such as H-ZSM-5 owing to their unique and yet versatile physical and chemical properties, which render not only high catalytic activity and product selectivity but also catalyst stability under harsh reaction conditions.^1^,^2^ Environmental processes like exhaust after-treatment utilise zeolites to adsorb/convert toxic pollutants, especially at the cold start of engines, which may originate from the unburnt fuel itself (*e.g.*, methanol and/or ethanol blended fuels^3^) or from the combustion process.^4^ The petro-chemical processes include conversion of methanol, which can be derived from renewable resources, into hydrocarbons (MTH) over H-ZSM-5. MTH promises to mitigate the growing global demand for gasoline, polymer grade olefins and aromatics through carbon neutral paths.^5^ Since its first development in 1976 (ref. 6 and 7) the potential impact of MTH on the energy and environment landscapes has stimulated interest in both industry and academia, and has triggered extensive research to understand the underlying reaction mechanism using experimental and computational tools.^1^,^8^–^16^ There is clear evidence that the catalytic activity arises from Brønsted acidic sites^1^,^8^,^9^,^12^ and that a hydrocarbon pool formed during the reaction in the zeolite pores plays an important role in the activity, product selectivity and zeolite lifetime.^17^ However, there is long standing uncertainty concerning the initial methoxylation, which is not only the first step in the process but also a key step in hydrocarbon (pool) growth through methylation.^1^,^8^,^9^,^18^–^22^ Methoxylation occurs on the reaction of methanol with Brønsted acidic sites (H^+^–O–Si/Al) of H-ZSM-5 giving rise to methoxy species,^8^,^12^,^18^,^19^,^21^,^22^ which is represented by:1
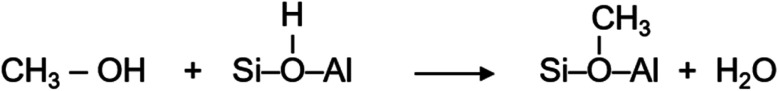



Besides methoxy species, water is a by-product whose formation is a clear indication of the methoxylation reaction. The direct role of methoxy species in the first C–C bond formation in the MTH reaction at 300 °C was recently reported by some of us.^23^ Moreover, the methylation (which is also known as alkylation) has an impact on a spectrum of petro- and fine- chemical processes.^18^–^26^

Studies based on solid state nuclear magnetic resonance (NMR) and infrared (IR) conclude that methoxylation occurs only at elevated temperatures (>150 °C).^22^,^27^,^28^ In line with these observations, several computational studies, usually examining configurations with only one methanol molecule per unit cell (*i.e.* one methanol molecule per Brønsted acidic site) suggest that methoxylation could have a significant activation energy (typically reported values are around 200 (±20) kJ mol^–1^) and hence require higher temperatures for the reaction.^15^,^19^–^22^ In a marked contrast, our recent inelastic neutron scattering (INS) and *operando* diffuse reflectance infrared Fourier transformed spectroscopy (DRIFTS) and mass spectrometry (MS) studies show the occurrence of methoxylation, to some degree, also under ambient conditions on H-ZSM-5 with a saturation level of methanol loading (7 molecules per Brønsted acidic site).^29^–^31^ Such a room temperature methoxylation reaction was indeed not ruled out in an earlier study.^32^ These observations are at least partially consistent with computational studies that report decreased energy barrier (reported values are varied; 130 (±30) kJ mole^–1^) for methoxylation with 2 methanol molecules per Brønsted acidic site, as compared to that with one methanol molecule.^20^,^33^–^36^ The calculated energy barrier, although greatly reduced, remains considerable for room temperature methoxylation.

No detailed experimental evidence on the role of methanol loading in methoxylation has been reported so far. The present study, therefore, evaluates the effect of the number of methanol molecules per acidic site on the methoxylation at room temperature by *operando* DRIFTS and MS. To this end, the methanol loading in H-ZSM-5 (Si/Al ≈ 25) pores is systematically varied between 32, 16, 8 and 4 molecules per unit cell, which corresponds to 8, 4, 2 and 1 molecules per Brønsted acidic site, respectively (Table 1). The results show that the higher the methanol loading, the faster the methoxylation. Accordingly, the reaction is more than an order of magnitude faster with 8 methanol molecules per Brønsted acidic site than that with 2 molecules. Significantly, we find that no methoxylation is observed with ≤1 molecule per acidic site.

**Table 1:** Methanol loading and assignment of observed vibrational frequencies for selected species

Methanol load	Observed vibrational Frequencies	Assignment		Reference
Molecules/acidic site	Wavenumbers (cm^–1^)	Vibrational mode	Structure	
8	870–880	*ν*_Si–O–Si_	Si–O–Si	63
	938 and 990	*ν*_C–O_	C–O	30, 64 and 65
	1180	*ρ*_CH_3__	Si/Al–O–CH_3_	30, 64 and 65
4	2868–2876	*ν*_sC–H_	Si/Al–O–CH_3_	23, 27 and 30
	2066	*ν*_sC–D_	Si/Al–O–CD_3_	12
2	2980–2973	*ν*_asC–H_	Si/Al–O–CH_3_	23, 27, 64 and 65
	2244	*ν*_asC–D_	Si/Al–O–CD_3_	12
	3611	*ν*_O–H_	Si/Al–O–H	23, 30, 63 and 65
1	3665	*ν*_O–H_	SiOH/Al–O–H	30, 63 and 65
	3725	*ν*_O–H_	X(–O–H)^*a*^	30 and 67
	3744	*ν*_O–H_	Si–O– H	23, 30, 63 and 65

^*a*^The structure is not known and hence the band is assigned to a differently coordinated –OH group.

## Methodology

### *Operando* diffuse reflectance infrared Fourier transformed spectroscopy (DRIFTS) and mass spectrometry (MS)

The zeolite H-ZSM-5 with a Si/Al ratio of 25, obtained from Zeolyst International, was calcined in air at 500 °C for 24 h. The BET surface area and total pore volume of the calcined zeolite are 390 m^2^ g^–1^ and 0.23 cm^3^ g^–1^, respectively. Mesopore surface area determined by t-plot method is 34 m^2^ g^–1^ indicating that the total surface area of the zeolite is mainly arising from micropores. *Operando* DRIFTS and MS experiments were conducted on an Agilent Cary 600 series spectrometer equipped with a Harrick Praying Mantis reaction cell that was connected to a gas dosing system.^30^ The reaction cell outlet was connected to a Hiden quantitative gas analysis (QGA) mass spectrometer for analysis of the products. Prior to the spectroscopic measurements, the zeolite was pre-treated in dry N_2_ flow (100 ml min^–1^) at 500 °C for a few hours and then cooled to room temperature (RT) under the same flow. Methanol pulse experiments were conducted under the same N_2_ flow at RT with different loadings that results in around 8, 4, 2 and 1 molecules per Brønsted acidic site.^29^–^31^ The evolution of surface adsorbed species and products were monitored by DRIFTS and MS, respectively, for around 30 min under the same N_2_ flush.

### Computational methods

Quantum mechanical/molecular mechanical (QM/MM) embedded calculations were employed to model zeolite H-ZSM-5. The ChemShell software^37^ was used to optimise zeolite geometries and determine vibrational frequencies of adsorbed species. For H-ZSM-5 models, the tetrahedral T12 site of the siliceous MFI was substituted with, Al which is located at the intersection of the sinusoidal channel. To form the Brønsted acidic site, a charge-compensating proton is bonded to the framework oxygen atom adjacent to T12 site^38^ and oriented towards the center of the super cage; this configuration presents the highest deprotonation energy *i.e.* most stable.^39^

For QM/MM calculations, the QM region is the chemically active part of the zeolite model and includes atoms up to the fifth oxygen atom (*i.e.* Al–O–Si–O–Si–O) from the central T12 site. The total number of atoms in the cluster model is 2165, including 74 QM atoms and 197 inner MM atoms. Atoms in the inner MM region can move during the geometry optimisation while, the outer MM region is frozen to ensure a bulk-like structure at a far limit from any chemical reactions. The inner and outer MM regions extend from the central T12 site to a radius of 10.58 Å (20 a_0_) and 21.17 Å (40 a_0_), respectively. The QM energy was calculated using spin unpolarised hybrid-DFT with the dispersion-corrected Becke97-3 exchange-correlation (XC) functional, B97-D, as provided in the GAMESS-UK code.^40^–^42^ The atomic orbitals were represented by the Ahlrichs and Taylor TZVP Gaussian basis sets.^43^ The self-consistent field (SCF) convergence criteria was set to an energy change of less than 2.72 × 10^–6^ eV (1 × 10^–7^ Hartrees) between SCF iterations.^44^,^45^ The MM energy was calculated using DL_POLY,^46^ employing the forcefield of Hill and Sauer,^47^,^48^ with the coordination dependent charges in the original forcefield being replaced with fixed 1.2 and –0.6 e point charges for silicon and oxygen, respectively^49^. Geometry optimisations (see ESI†) were performed in a Cartesian coordinate space using the Limited-Memory Broyden-Fletcher-Goldfarb-Shanno (L-BFGS) algorithm, with a maximum gradient convergence threshold of 0.015 eV Å^–1^.^50^–^53^

Vibrational frequencies were calculated using ChemShell, with a task-farmed finite-difference approach,^40^ allowing us to confirm that geometries correspond to local minima.^54^,^55^ For the vibrational frequency calculations, the adsorbate, active site and second neighbouring framework atoms were displaced; comparison of this approximation against the displacement of all atoms in the QM region shows no differences, as previously reported.^56^ Because our simulations aimed to calculate mainly vibrational frequencies of the CH_3_ group, a scaling factor for the computed vibrational frequencies was calculated using data for the CH_3_ asymmetric stretch of methanol at 93 K, which is the lowest experimental temperature reported.^57^ The resulting scaling factors of 0.9306 and 0.9553 were used for normal and deuterated methanol, respectively. The scaling factors are well within the range previously employed of between 0.9 and 0.9614.^58^–^61^

## Results and discussion

### *Operando* DRIFTS and MS studies

**Fig. 1:**
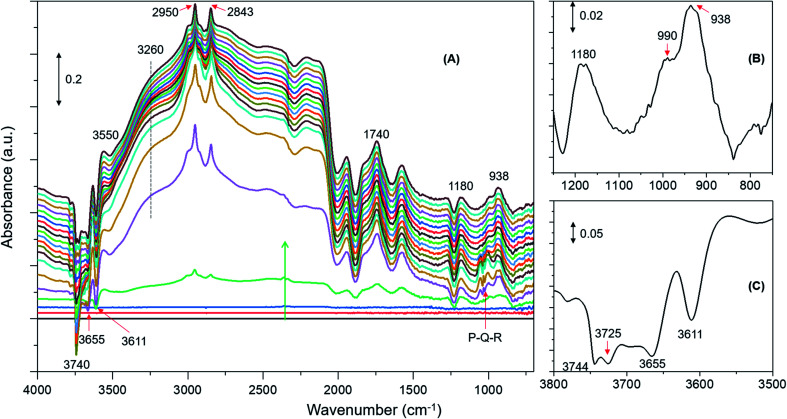
Infrared difference spectra of zeolite H-ZSM-5 with a methanol loading of 8 molecules per Brønsted acidic site at room temperature. Magnified regions of a representative spectrum are shown (B) and (C). Refer to Experimental section for details.

DRIFTS difference spectra of zeolite H-ZSM-5 with a methanol loading of 8 molecules per acidic site are shown in Fig. 1. It is evident that the spectra are dominated by hydrogen bonded methanol species that are unambiguously characterised by the triplet arising from the Fermi resonance caused by *ν*(O–H) (of both methanol and zeolite OH groups) with 2*δ* and 2*γ* overtones, which falls between 1500 and 3500 cm^–1^.^62^,^63^ Within this region, the C–H stretching modes of the hydrogen bonded methanol (protonated methanol geometry and is discussed later) between 3100 and 2800 cm^–1^ are evident; however, no other bands attributable to methoxy species could be distinguished. Therefore, wavenumbers below 1500 and above 3500 cm^–1^ are probed for evidence of methoxy and O–H stretching modes, respectively.^30^ A band at 938 that contains a shoulder at 990 is present along with another band at 1180 cm^–1^ (Fig. 1B). Typical P–Q–R bands of gas phase methanol appear at around 1008, 1032 and 1056 cm^–1^ (Fig. 1A); however, these bands disappear within the first few minutes of the reaction under the N_2_ flow. In the *ν*(O–H) region, at least four negative bands appear between 3600 and 3750 cm^–1^ (Fig. 1C and Table 1). The band at 938 cm^–1^ is attributed to C–O stretch of the methoxy species^30^,^64^ and the corresponding methyl rock band appears at 1180 cm^–1^ (ref. 65) indicating the occurrence of methoxylation at RT.^30^ The shoulder at 990 cm^–1^ could also arise from the C–O stretch of a second type of methoxy species or of the hydrogen bonded methanol.^30^ In line with the low frequency methoxy bands, consumption of different hydroxyls in the reaction (either in hydrogen bonding or methoxylation) is evident from negative bands above 3600 cm^–1^ (Fig. 1A and C). The negative bands at 3610 and 3744 cm^–1^ are due to consumption of Brønsted acidic and silanol groups, respectively.^30^,^62^,^63^ The band at 3665 cm^–1^ was previously assigned to hydrolysed extra-framework Al(Al–OH).^30^,^63^ The assignment of the band at around 3725 cm^–1^ is not straight forward and hence it is tentatively attributed to the involvement of differently coordinated hydroxyl groups.^30^

**Fig. 2:**
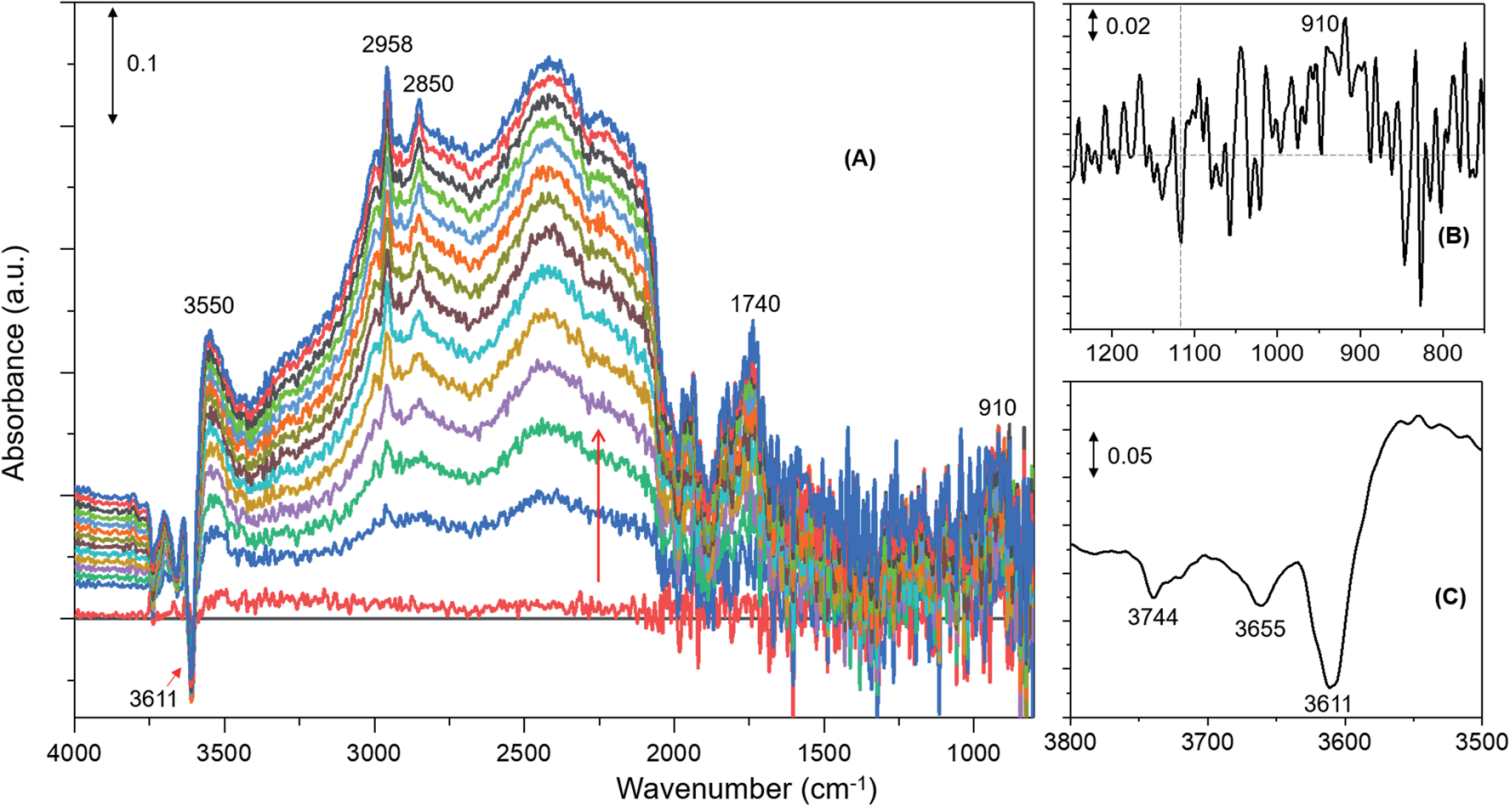
Infrared difference spectra of H-ZSM-5 with a methanol loading of 1 molecule per Brønsted acidic site at room temperature. Magnified regions of a representative spectrum are shown (B) and (C).

**Fig. 3:**
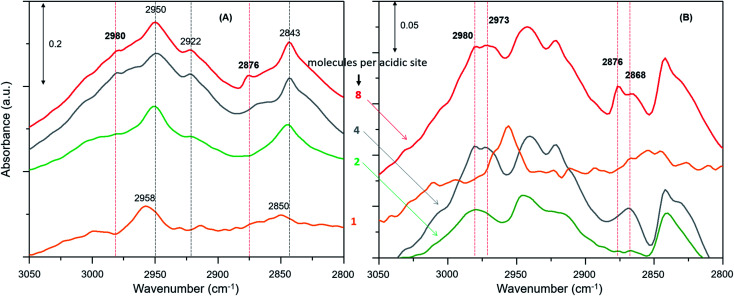
(A) Magnified infrared difference spectra of H-ZSM-5 with different methanol loadings at room temperature and (B) comparison of difference spectra between the earliest and at a later stage of the reaction.

Similar spectroscopic features are observed for zeolites with methanol loadings of 4 and 2 molecules per Brønsted acidic site, although the overall intensity of the spectra is reduced by decreasing methanol loading from 8 to 2 molecules per acidic site. However, striking differences in the consumption of hydroxyls (above 3600 cm^–1^) and low frequency methoxy bands (below 1500 cm^–1^) are evident from the zeolite with the lowest methanol loading of 1 molecule per acidic site (Fig. 2) as compared to that with the highest methanol loaded zeolite (Fig. 1). Examining the DRIFTS spectra of the lowest methanol loading in more detail, we note first that the signature infrared profile of the lowest methanol loaded zeolite in Fig. 2 matches precisely that of hydrogen bonded methanol with a neutral geometry,^32^,^62^ which is different from the highest methanol loaded zeolite profile (Fig. 1) that reflect the protonated methanol geometry.^30^,^32^,^62^ The C–H stretching modes of the neutral geometry appear at 2958 and 2850 cm^–1^ which can be attributed to *ν*_as_(C–H) and *ν*_s_(C–H) modes, respectively, of hydrogen bonded methanol with the neutral geometry.^62^,^63^ The position of the bands is at slightly higher wavenumbers as compared to that of the protonated geometry which shows up at 2950 and 2843 cm^–1^ (Fig. 1), in line with previous studies.^30^,^63^ The band at 3550 cm^–1^ is also characteristic of the hydrogen bonded methanol with a neutral geometry^62^ and the band intensity increases with increasing hydrogen bonded methanol triplet and with a growing negative band at 3610 cm^–1^, which is indicative of the involvement of Brønsted acidic sites. Moreover, the absence of multiple hydrogen bonded methanol molecules in the form of dimers or oligomers is evident when we compare Fig. 1 and 2 from the missing absorbance centred at 3260 cm^–1^.^62^,^63^ Even more significantly, only one negative band at 3611 cm^–1^ is present, unlike four negative bands for the highest methanol loaded zeolite, and bands assignable to low frequency methoxy bands are completely missing. Instead, a broad band between 1000 and 850 cm^–1^ is observed, which could be a combination of different bands. For example, a contribution from the Si–O–Si asymmetric stretch at around 880 cm^–1^ (Fig. 2B and Table 1) and hydrogen bonded methanol bands (assignable to C–O stretch and methyl rock) could also contribute between 900 and 1100 cm^–1^. Accordingly, the negative band at 3610 cm^–1^ is attributed to involvement of Brønsted acidic hydroxyls in hydrogen bonding with methanol indicating that the Brønsted acidic hydroxyls are more reactive than other hydroxyls such as hydrolysed extra-framework Al–OH and silanol groups (see Fig. 1).^30^ It is noteworthy that the methyl rock of methoxy band at around 1180 cm^–1^ is not present and hence a contribution from the C–O stretch of the methoxy species to the broad band between 1100 and 850 cm^–1^ can be ruled out, which is further indicated by the signature C–H stretching modes of the methoxy and hydrogen bonded methanol. To this end, the C–H stretching region is magnified and four different methanol loading experiments are compared in Fig. 3.

**Fig. 4:**
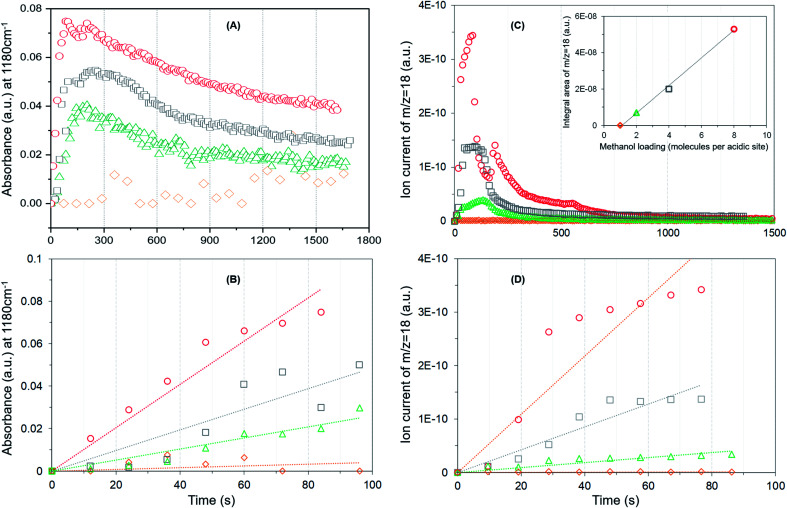
The evolution of methoxy species reflected by the band at 1180 cm^–1^ (A), rate of methoxy formation (B), water formation (C) and rate of water formation (D) as a function of time. First 100 s of the reaction is considered for the rates. Methanol loading of 8 (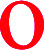), 4 (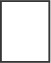), 2 (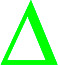) and 1 (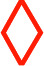) molecules per acidic site. Inset in (C) compares the area under the MS profile as a function of methanol loading.

The zeolite with the highest methanol loading of 8 molecules per acidic site clearly shows bands at 2980 and 2876 cm^–1^, attributable to *ν*_as_(C–H) and *ν*_s_(C–H) of methoxy species (Fig. 3A).^27^,^30^,^62^,^63^,^65^ However, these bands are somewhat obscured by the intense triplet arising from the protonated hydrogen bonded methanol species that present C–H stretching bands at 2950, 2922 and 2843 cm^–1^.^30^,^62^,^63^,^65^ It appears that the intensity of the methoxy bands at 2980 and 2876 cm^–1^ diminishes on decreasing the methanol loading from 8 to 2 molecules per acidic site, and with no indication of such bands for the lowest methanol loading of 1 molecule per acidic site. To verify this, the difference spectra derived from the earliest measurement and at a later stage of the reaction are compared in Fig. 3B. It is clear that the bands assignable to methoxy species emerge at 2980 and 2876 cm^–1^, and 2973 and 2868 cm^–1^,^8^,^23^,^27^,^30^ which imply the occurrence of at least two types of methoxy species. This observation is consistent with our earlier INS and DRIFTS data that show two types of methoxy species^29^,^30^ and is in line with earlier infrared and NMR reports.^27^,^28^ Clearly, the intensity of the methoxy bands at 2980 and 2875 cm^–1^, and 2973 and 2865 cm^–1^ diminishes on decreasing the methanol loading from 8 molecules per acidic site to 2 and no bands attributable methoxy species are present for the lowest methanol load of 1 molecule per acidic site (Fig. 3B), suggesting the occurrence of loading dependent room temperature methoxylation. This conclusion is further corroborated by the evolution of the methyl rock band of methoxy species at 1180 cm^–1^ (Fig. 4).

It is evident from Fig. 4A that the methyl rock band grows rapidly for the highest methanol loading zeolite and thereafter decreases gradually suggesting the occurrence of partial hydrolysis of methoxy species due to the presence of water, which is a result of the methoxylation reaction (eqn (1)), in the zeolite pores.^30^ A similar observation is also reported by NMR and, significantly those methoxy species on ZSM-5 are not completely eroded on hydrolysis with water at room temperature unlike on Y and SAPO-34,^28^ implying the unique intrinsic nature of active acidic sites in ZSM-5. The evolution of the band is clearly hampered on decreasing the methanol loading from 8 to 2 molecules per acidic site and crucially no intensity gain of the band is observed for the lowest methanol loading of 1 molecule per acidic site (Fig. 4A). Based on this observation, the rate of methoxylation as a function of methanol loading is derived by following the rate of intensity gain of the methyl rock band at 1180 cm^–1^ (Fig. 4B), for which the evolution of the band during the first 100 seconds is considered. The rate of methoxylation is 20 and 40 times faster for the zeolite with the highest methanol loading of 8 molecules per acidic site than that for the loadings of 4 and 2 molecules per acidic site, respectively. The occurrence of room temperature methoxylation is further corroborated with MS data (Fig. 4C) which show the evolution of water during the reaction. It is evident that the relative amount of water formed during the reaction decreases on decreasing the methanol loading from 8 to 2 molecules per acidic site and no water formation is observed for the lowest methanol loading of 1 molecule per acidic site, which is confirmed by the area under the MS profile as a function of methanol loading in the inset of Fig. 4C. The rate of water formation is derived from the first 100 seconds of the reaction (Fig. 4D), which is similar to that considered for the rate of methyl rock band evolution (Fig. 4B). As expected, the rate of water formation is 2 and 12 times faster for the highest methanol loading zeolite than that for the 4 and 2 molecules per acidic sites, respectively. The relative rates derived from DRIFTS and MS are at least an order of magnitude faster for the highest methanol loading zeolite than that for the zeolite with a loading of 2 molecules per acidic site and confirm the loading dependent room temperature methoxylation in zeolite H-ZSM-5.

**Fig. 5:**
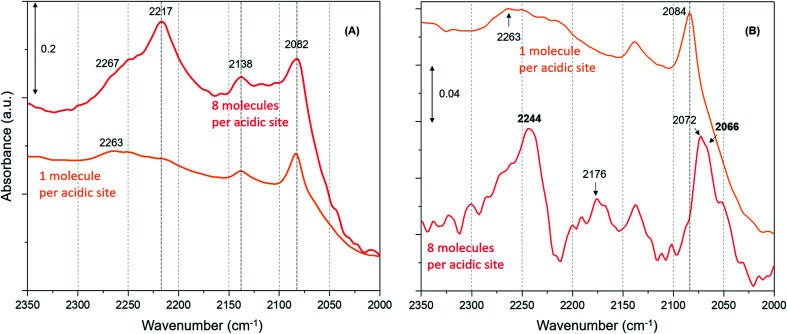
(A) Magnified infrared difference spectra of H-ZSM-5 with different CD_3_OH loadings at room temperature and (B) compares difference spectra between the earliest and at a later stage of the reaction.

Furthermore, we have verified the occurrence of loading dependent room temperature methoxylation by isotopic methanol experiments using CD_3_OH. Zeolites with loadings of 8 (the highest) and 1 (the lowest) methanol molecules per acidic site are compared in Fig. 5. The hydrogen bonded methanol C–D stretching bands at 2217 and 2082 cm^–1^ attributable to *ν*_as_(C–D) and *ν*_s_(C–D) modes are present for both the zeolites (Fig. 5A). However, no bands assignable to methoxy C–D stretching modes are visible from Fig. 5A and hence difference spectra derived from the earliest measurement and at a later stage of the reaction are compared in Fig. 5B, which is similar to the one reported in Fig. 3B. It is evident that the highest methanol loading zeolite exhibits the bands that are attributable to methoxy species at around 2242, 2176 and 2066 cm^–1^,^12^ while these bands are completely missing for the lowest methanol loading zeolite. The bands at 2242 and 2176 cm^–1^ are assigned to methoxy *ν*_as_(C–D) and the corresponding *ν*_s_(C–D) stretching modes are at 2072 and 2066 cm^–1^, which appear to encompass two types of methoxy species. The band at 2072 cm^–1^ should be treated with some caution because the hydrogen bonded methanol with neutral (lowest methanol load) geometry exhibits a blue shift in the C–D stretching modes as compared with the protonated geometry (highest methanol load), as discussed for the normal methanol loading experiments in Fig. 1 and 2. Thus, the band at 2072 cm^–1^ could also reflect the hydrogen bonded methanol of protonated geometry, whilst the counterpart band at 2082 cm^–1^ is observed for the neutral geometry. Nonetheless, the isotopic methanol experiments confirm the occurrence of loading dependent room temperature methoxylation, in agreement with normal methanol loading experiments reported in Fig. 3B and also in agreement with our earlier INS and DRIFTS studies.^29^,^30^ In line with these observations, water formation is observed only for the highest methanol loading zeolite by MS (not shown), and no water formation is detected for the lowest methanol loading zeolite, consistent with the above results reported with normal methanol experiments in Fig. 4C. In line with methanol loading dependent room temperature methoxylation experiments, recent simulations of methoxylation show that the energy barriers decrease from 160 to 119 kJ mol^–1^ when the loading is increased from 1 to 4 methanol molecules per Brønsted acidic site, respectively.^66^ The energy barriers calculated are in line with the previous literature.^20^,^33^–^36^ However, the energy barrier of 119 kJ mol^–1^ for 4 methanol molecules per acidic site remains significant in the context of a process observed at room temperature. Also, we recognise the importance of Si/Al ratio of the zeolite in the reaction which is a topic for future study.

### Computational studies

To complete our analysis, the assignment of the DRIFTS bands to surface methoxy and hydrogen bonded methanol species is verified by quantum mechanical/molecular mechanical (QM/MM) calculations (Table 2 and 3). Calculated vibrational frequencies of methoxy species at the Brønsted acidic site are 925, 1157/1165, 2880 and 2988 cm^–1^. The band at 925 cm^–1^ is arising from the *ν*(C–O) stretch (structure A in Table 2)^21^ and the pair of bands at 1157/1165 cm^–1^ are due to perpendicular and parallel methyl rock modes (the parallel mode is represented by structure B in Table 2), which themselves are not resolved in the DRIFTS data (see Fig. 1 and ref. 30). The bands at 2880 and 2988 cm^–1^ result from *ν*_s_(C–H) and *ν*_as_(C–H) stretching modes of the methoxy species, respectively (structures C and D in Table 2). By comparison with the DRIFTS data, the calculated vibrational frequencies of methoxy *ν*(C–O) stretch and methyl rock modes are slightly red shifted, while *ν*(C–H) stretching modes are slightly blue shifted. Also, *ν*(C–O) stretch and methyl rock modes are slightly red shifted as compared to those reported in our earlier study, which can be attributed to the two different methods employed.^30^ The calculated vibrational frequencies of the methoxy species are slightly affected by co-adsorption of either methanol and/or water molecules in the unit cell, representing the experimental conditions, which indicates the sensitivity of vibrational frequencies of adsorbed species to the local environment of the zeolite unit cell. Based on these observations, we conclude that the calculated vibrational frequencies are in line with the experimental DRIFTS data as is evident from Table 1 and 2. The vibrational frequencies of deuterated methoxy species are calculated to be 753, 865/900, 2078 and 2253 cm^–1^, which are arising from *ν*(C–O) stretch, *ρ*(CD_3_) rock, *ν*_s_(C–D) and *ν*_as_(C–D) modes, respectively (Table 2). The calculated *ν*(C–D) stretching modes represent an ideal case of only one kind of methoxy species as opposed to the experimental data that reflect a complex combination of reactants and product molecules in a unit cell, and hence are blue shifted slightly as compared to the DRIFTS results (see Table 1 and 2).

**Table 2:** Calculated methoxy vibrational frequencies are shown for selected modes with their structures

Structure	Vibrational frequencies, (cm^–1^)	Vibrational mode	Reference
A	925	*ν*_C–O_(Si/Al–OCH_3_)	21 and 30
	753	*ν*_C–O_(Si/Al–OCD_3_)	
B	1157/1165	*ρ*_CH_3__OCH_3_	30
	865/900	*ρ*_CH_3__OCD_3_	This work
C	2880	*ν*_sC–H_(Si/Al–O–CH_3_)	This work
	2078	*ν*_sC–D_(Si/Al–O–CD_3_)	
D	2988	*ν*_asC–H_(Si/Al–O–CH_3_)	This work
	2253	*ν*_asC–D_(Si/Al–O–CD_3_)	
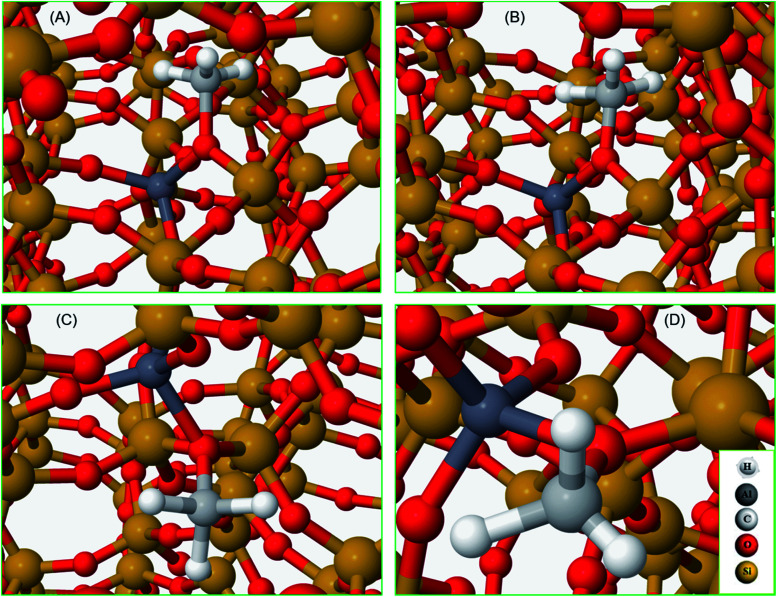			

**Table 3:** Experimental and calculated vibrational frequencies of hydrogen bonded methanol are compared for selected C–H stretching modes with their structures. In the accompanying figure, some atoms have been removed to make visualisation easier for the reader

Structure	Experimental (cm^–1^)	Calculated (cm^–1^)	Assignment, vibrational mode	Reference
**Neutral geometry**				
(A)	2850	2848	*ν*_sC–H_	14, 32, 62, 63, 58 and 68–70
	2082	2093	*ν*_sC–D_	
	2958	2962	*ν*_asC–H_	
	2217/2250/2263	2262	*ν*_asC–D_	

**Protonated geometry**				
(B)	2843	2844	*ν*_sC–H_	14, 32, 62, 63, 58 and 68–70
	2082	2083	*ν*_sC–D_	
	2950	2944	*ν*_asC–H_	
	2217/2250/2267	2272	*ν*_asC–D_	
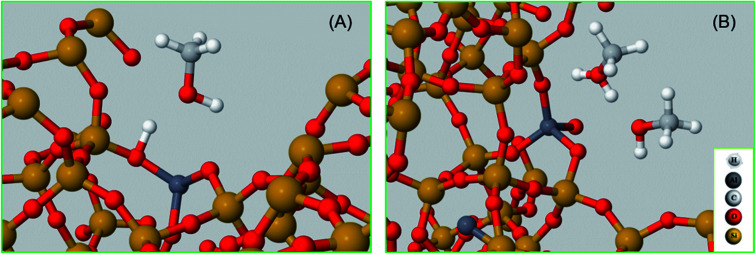				

The vibrational frequencies of hydrogen bonded methanol with neutral and protonated geometries are also calculated and compared with the experimental data in Table 3. Addition of one methanol molecule at the Brønsted acidic site gives rise to hydrogen bonded structure with the neutral geometry as depicted with structure A in Table 3. The geometry forms a six membered ring structure at the Brønsted acidic site and is similar to the one reported in the literature.^14^,^32^,^62^ The calculated vibrational frequencies for the *ν*_s_(C–H) and *ν*_as_(C–H) stretching modes of the neutral geometry are 2848 and 2962 cm^–1^, respectively, and are in excellent agreement with the experimental data reported in Table 3 and, Fig. 3 and 5. The corresponding deuterated methanol geometry gives rise to vibrational frequencies of 2093 and 2262 cm^–1^ for *ν*_s_(C–D) and *ν*_as_(C–D) stretching modes, which are in line with DRIFTS data that show the *ν*_s_(C–D) mode at 2082 cm^–1^ and the broad band that envelops the *ν*_as_(C–D) region with an indication at 2263 cm^–1^. Addition of a second methanol molecule in the unit cell results in an eight membered ring structure at the Brønsted acid site and that the acidic proton shuttles between the framework oxygen and methanol hydroxyl, forming a protonated methanol geometry as depicted with structure B in Table 3. The vibrational frequencies calculated for the *ν*_s_(C–H) and *ν*_as_(C–H) stretching modes of the protonated methanol geometry are calculated to have frequencies of 2844 and 2944 cm^–1^, respectively. These values are consistent with the experimental observations (Table 3), including the observed relative redshift in the vibrational frequencies of *ν*(C–H) modes of the neutral geometry as compared with the protonated one.^63^ The experimental results show *a* ≈8 cm^–1^ redshift for the neutral geometry as compared to protonated geometry, while simulations show 10 (±5) cm^–1^. The corresponding deuterated methanol geometry yields vibrational frequencies of 2083 and 2272 cm^–1^ for *ν*_s_(C–D) and *ν*_as_(C–D) modes. The vibrational frequency of 2083 cm^–1^ for *ν*_s_(C–D) is in excellent agreement with the observed DRIFTS band, whereas the calculated 2272 cm^–1^ frequency for *ν*_as_(C–D) appeared as a shoulder at 2267 cm^–1^ to the prominent band at 2217 cm^–1^ observed by DRIFTS. This once again suggests the occurrence of a combination of different bands in the *ν*_as_(C–D) region under experimental conditions, which might be difficult to be captured completely by simulations.^67^ Nonetheless, the calculated vibrational frequencies do reflect our assignment of the DRIFTS bands as is evident from Table 1, 2 and 3.

## Summary and conclusions

Our study of the methanol loading dependent methoxylation in zeolite H-ZSM-5 (Si/Al ≈ 25) pores under ambient conditions has enabled us to probe simultaneously methoxy species and reaction products. The assignments of infrared vibrational frequencies of methoxy and hydrogen bonded methanol have been supported by QM/MM simulations, which are consistent with the experimental DRIFTS data. Both experiment and simulation show that the methoxy bands at around 940, 1180, 2868–2876 and 2980–2973 cm^–1^ correspond to *ν*(C–O), *ρ*(CH_3_), *ν*_s_(C–H) and *ν*_as_(C–H), respectively.

From our results, it is evident that the room temperature methoxylation in H-ZSM-5 is methanol loading dependent: the higher the loading, the faster the methoxylation. The reaction is more than an order of magnitude faster with 8 molecules per Brønsted acidic site than that with 2 molecules. As well as methoxylation, hydrogen bonded methanol with protonated geometries are also formed. Significantly, no methoxylation is observed with methanol loading of ≤1 molecule per acidic site, but only hydrogen bonded methanol with neutral geometry is detected. Thus, the structure of hydrogen bonded methanol is also loading dependent. These findings will have significant implications for reactions involving zeolite H-ZSM-5 and methanol.

## Conflicts of interest

There are no conflicts to declare.

## Supplementary Material

Supplementary information
