# Supplementary material for: Methanol loading dependent methoxylation in zeolite H-ZSM-5
Source: Chem Sci. 2020 Jun 17;11(26):6805–14. doi: 10.1039/d0sc01924k (PMC7448526; doi:10.1039/d0sc01924k)
Supplement: Supplementary file 1 [file SC-011-D0SC01924K-s001.pdf]

Electronic Supplementary Material (ESI) for Chemical Science.
This journal is © The Royal Society of Chemistry 2020

Supplementary Information:

Methanol Loading Dependent Methoxylation in zeolite H-ZSM-5

Santhosh K. Matam*[1,2] Stefan Nastase[1,2], Andrew Logsdail[1,2] and C. Richard. A Catlow[1,2,3]

[1]UK Catalysis Hub, Research Complex at Harwell, Science and Technology Facilities Council, Rutherford Appleton Laboratory, Oxford, OX11 0FA, UK

[2]Cardiff Catalysis Institute, School of Chemistry, Cardiff University, Cardiff, CF10 3AT UK.

[3]Department of Chemistry, University College London, 20 Gordon St., London WC1E 6BT, UK

E-mail: santhosh.matam@rc-harwell.ac.uk; website: http://www.ukcatalysishub.co.uk/

Geometric analysis:

A series of geometric observables, illustrated in Figure 1, were determined in order to analyse any potential correlation between specific bond distances and their corresponding vibrational frequencies.

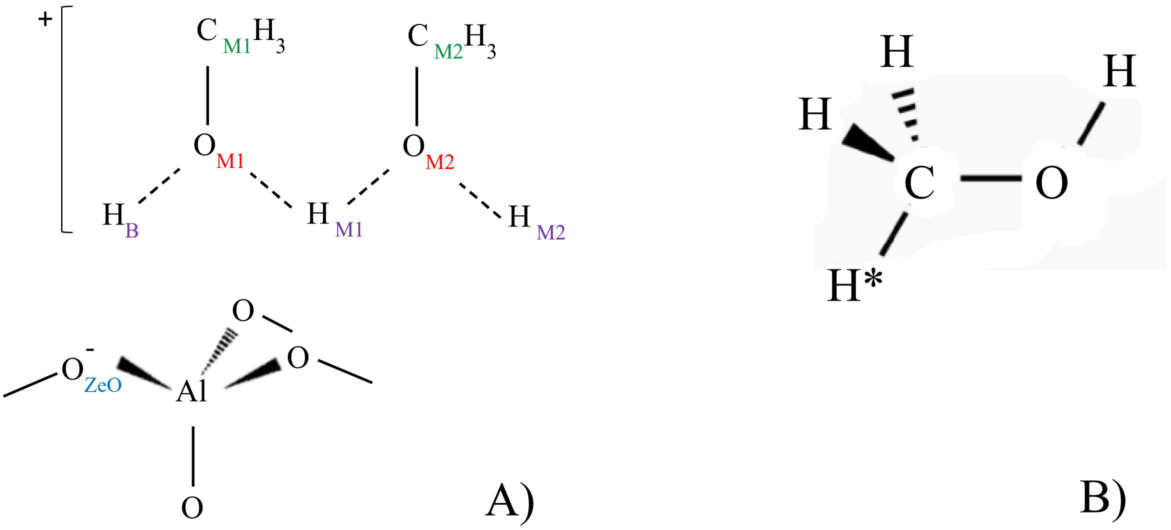

A) B)

Figure S1. Schematic representation of analysed geometric observables of A) bi-methanol adsorbed on the zeolite Brønsted acid site and B) gas phase methanol, with the presented indices highlighting "B" – Brønsted proton of the acid site, "M1" – atom of the closest methanol to the acid site or of the methoxy group, "M2" – atom of the co-adsorbed methanol molecule and "*" indicating the hydrogen atom of the methyl group placed in an in the equatorial position with respect to the hydrogen atom of OH group in the case of a methanol adsorbed model or Aluminium of the active site when considering the zeolite bound methoxy case.

A detailed presentation of the bond distances is provided in Table S1. To focus on the methyl vibrational modes, the correlation between the C-H and C-O bonds with the symmetric and asymmetric vibrational frequencies of the methyl group was analysed. As shown in Table S1, the variation of the C_{M1}-H* bond distance was similar to that of C_{M1}-H, thus only the C_{M1}-H* bond distance was considered for analysis. However, based on Figs. 1 and 2, no linear correlation is found between the C-O and C-H bond distances and the symmetric and asymmetric vibrational frequencies of the methyl group.

Table S1. Summary of bond distances illustrated in Figure 1 of protonated methanol (CH_3OH^+) of bi-methanol model, neutral methanol (CH_3OH) of single methanol model, and zeolite bound methoxy group (Zeo-CH_3), presented in Ångstroms.

Model	O_{Zeo}-H_B	O_{M1}-H_B	O_{M1}-H_{M1}	O_{M2}-H_{M1}	O_{M2}-H_{M2}	
CH_3OH^+	1.676	1.001	1.044	1.445	0.961	
CH_3OH	1.046	1.479	0.965			
	C_{M1}-O_{M1}	C_{M2}-O_{M2}	C_{M1}-H*	C_{M1}-H	C_{M2}-H*	C_{M2}-H
CH_3OH^+	1.457	1.442	1.084	1.085	1.085	1.089
CH_3OH	1.437		1.086	1.089		
Zeo- CH_3	1.484		1.083	1.084		

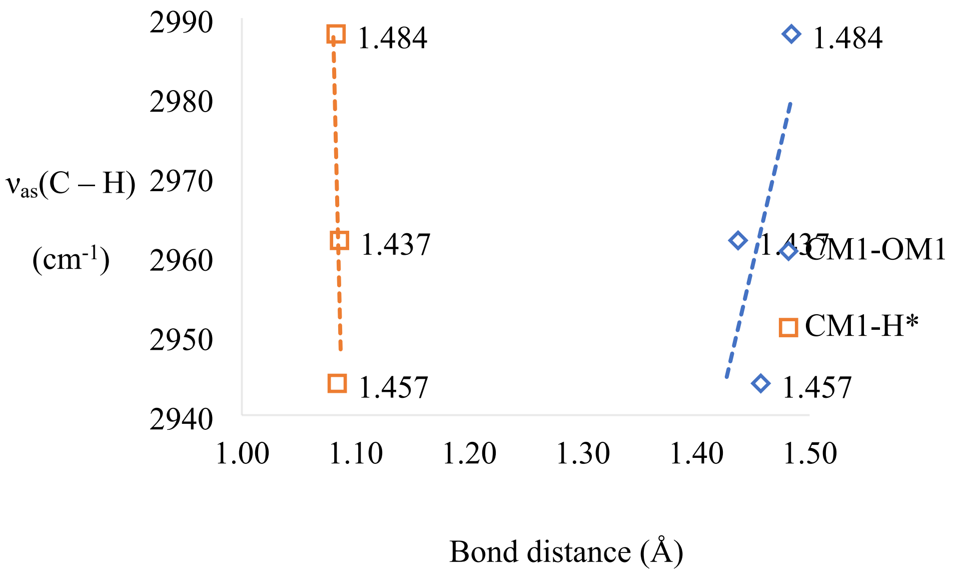

Figure S2. Plot between the C_{M1}-O_{M1} and C_{M1}-H* bond distances and the asymmetric vibrational frequency of methyl, presented in Ångstroms and cm^{-1}.

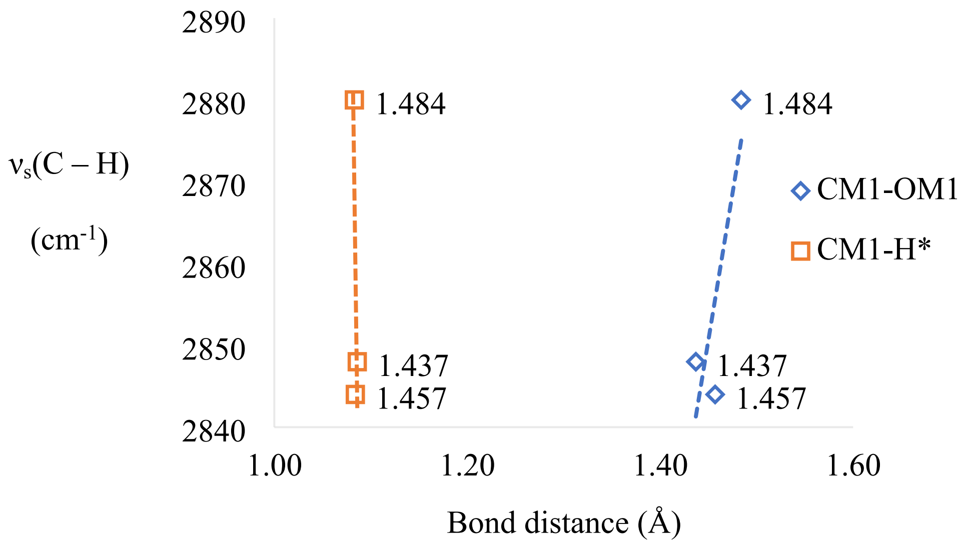

Figure S3. Plot between the C_{M1}-O_{M1} and C_{M1}-H* bond distances and the symmetric vibrational frequency of methyl, presented in Ångstroms and cm^{-1}.
